# *Cis*-Palmitoleic Acid Regulates Lipid Metabolism via Diacylglycerol Metabolic Shunting

**DOI:** 10.3390/foods14142504

**Published:** 2025-07-17

**Authors:** Wenwen Huang, Bei Gao, Longxiang Liu, Qi Song, Mengru Wei, Hongzhen Li, Chunlong Sun, Wang Li, Wen Du, Jinjun Shan

**Affiliations:** 1 College of Biological and Pharmaceutical Engineering, Shandong University of Aeronautics, Binzhou 256603, Chinasongqi@sdua.edu.cn (Q.S.); hongzhenli2025@126.com (H.L.);; 2 School of Marine Sciences, Nanjing University of Information Science and Technology, Nanjing 210023, China; bgao@nuist.edu.cn (B.G.);; 3 Jiangsu Key Laboratory of Children’s Health and Chinese Medicine, Nanjing University of Chinese Medicine, Nanjing 210023, China

**Keywords:** *cis*-palmitoleic acid (*c*POA), diacylglycerol (DAG) shunting, obesity, metabolic reprogramming, phospholipid synthesis

## Abstract

Obesity and related metabolic disorders are closely linked to dysregulated lipid metabolism, where the metabolic balance of diacylglycerol (DAG) played a pivotal role. Although *cis*-palmitoleic acid (*c*POA) exhibits anti-obesity effects, its efficacy varies across dietary conditions, and its molecular mechanisms remains unclear. In this study, we investigated the dose-dependent regulatory effects of *c*POA on DAG metabolic shunting in db/db mice, employing lipidomics, pathway analysis, and gene/protein expression assays. Under a basal diet, low-dose *c*POA (75 mg/kg) inhibited DAG-to-triglyceride (TAG) conversion, reducing hepatic lipid accumulation, while medium-to-high doses (150–300 mg/kg) redirected DAG flux toward phospholipid metabolism pathways (e.g., phosphatidylcholine [PC] and phosphatidylethanolamine [PE]), significantly lowering body weight and adiposity index. In high-fat diet (HFD)-fed mice, *c*POA failed to reduce body weight but alleviated HFD-induced hepatic pathological damage by suppressing DAG-to-TAG conversion and remodeling phospholipid metabolism (e.g., inhibiting PE-to-PC conversion). Genetic and protein analyses revealed that *c*POA downregulated lipogenic genes (SREBP-1c, SCD-1, FAS) and upregulated fatty acid β-oxidation enzymes (CPT1A, ACOX1), while dose-dependently modulating DGAT1, CHPT1, and PEMT expression to drive DAG metabolic shunting. Notably, DAG(36:3, 18:1–18:2) emerged as a potential biomarker for HFD-aggravated metabolic dysregulation. This study elucidated *c*POA as a bidirectional regulator of lipid synthesis and oxidation, improving lipid homeostasis through dose-dependent DAG metabolic reprogramming. These findings provide novel insights and strategies for precision intervention in obesity and related metabolic diseases.

## 1. Introduction

Obesity and its associated metabolic disorders, such as type 2 diabetes, non-alcoholic fatty liver disease (MASLD), and cardiovascular complications, have reached epidemic proportions globally, driven by excessive lipid accumulation and disrupted energy homeostasis [1,2]. Central to these pathologies was the dysregulation of lipid metabolism, particularly the imbalance between triglyceride (TAG) synthesis and lipolysis [3]. Diacylglycerols (DAGs), key intermediates in lipid metabolism, played dual roles in energy storage and cellular signaling [4]. While DAG accumulation was linked to insulin resistance and hepatic steatosis [5], evidence suggested that redirecting DAG flux toward phospholipid synthesis—rather than TAG storage—may mitigate metabolic dysfunction [6,7]. However, the mechanisms underlying metabolic reprogramming and its therapeutic potential remained poorly understood.

*Cis*-palmitoleic acid (*c*POA), a monounsaturated fatty acid that naturally present in fish oils and plant oil, has garnered attention for its anti-obesity and insulin-sensitizing effects [8]. Unlike other fatty acids, *c*POA was a major component of human adipose tissue and acted as a lipid signaling molecules to regulate lipid metabolism [9]. However, its specific molecular targets and metabolic pathways remained unclear. Previous studies found that under basal dietary conditions, *c*POA reduced body weight, decreased fat accumulation, and repaired liver injury, but exhibited paradoxical dose-dependent effects. In contrast, under high-fat diet (HFD) conditions, its weight-reducing effect was not significant, while its liver-repairing effect was enhanced [10]. It remained unclear whether the HFD masked the effects of *c*POA or whether other factors were responsible. Research demonstrated that DAG served not only as a precursor for phospholipid synthesis [11], but also as the foundation for TAG formation [3]. Importantly, the fate of DAG—whether it was incorporated into phospholipid membranes or lipid droplets—could determine its metabolic consequences [12]. We postulated that DAG metabolism might be the key of *c*POA action; nevertheless, no study had systematically investigated how *c*POA affected DAG metabolic pathways.

Here, we employed db/db mice as the experimental animal model to investigate the dose-dependent effects of *c*POA on hepatic DAG metabolism. The db/db mouse model replicates the genetic susceptibility background of human obesity-associated MASLD [13]. HFD can further exacerbate hepatic lipotoxic injury in db/db mice. This “genetic + environmental dual-hit strategy” more closely recapitulates the clinical phenotype of obese patients with diet-aggravated metabolic deterioration. By integrating lipidomics and pathway analysis, we systematically mapped *c*POA’s influence on DAG metabolic shunting across different doses. Furthermore, we compared metabolic pathway alterations between basal and HFD conditions to uncover diet-dependent regulatory mechanisms. To elucidate key regulatory nodes, we validated critical enzymes in DAG metabolic shunting, along with their upstream and downstream effectors, at both transcriptional and protein levels. Our findings demonstrate that *c*POA exhibits a context-dependent therapeutic window, functioning as a versatile modulator of lipid metabolic flexibility. To the best of our knowledge, this study uncovers a novel mechanism of *c*POA-mediated lipid metabolism and proposes a targeted intervention strategy for DAG-driven lipid regulation.

## 2. Materials and Methods

### 2.1. Experimental Animals

The db/db mice model was used in this study. These mice are leptin receptor (Lepr)-deficient and characterized by disordered lipid metabolism, obesity and diabetes. In female db/db mice, the masses of the uterus and ovaries are low, and the secretion of female sex hormones is reduced. Consequently, these homozygous mice are sterile [14]. The mice were maintained on a Dock7m background, which exhibits normal body weight and fat content; therefore, db/m mice are commonly used as controls. Db/db mice (specific pathogen-free, males, 15 weeks of age) and db/m mice were purchased from Changzhou Cavens Laboratory Animal Co., Ltd. (Jiangsu, China).

The mice were multiple-housed in filter-top cages at 23 ± 2 °C, with a 12 h light/dark cycle. They had ad libitum access to food and water throughout the study period. At the end of the study, they were euthanized by cervical dislocation. All animal experiments were performed in accordance with the guidelines of Nanjing University of Chinese Medicine for the ethical care of animals and were approved by the Medical Ethics Committee of Nanjing University of Chinese Medicine. The study was conducted in accordance with the National Institutes of Health Guide for the Care and Use of Laboratory Animals (NIH Publications No. 8023, revised 1978).

### 2.2. Animal Groups and Diets

In the first experiment, following one-week acclimation period, 10 db/m mice and 40 db/db mice were maintained on a basal diet. The body weight of each mouse was measured and the mice were randomly allocated to the following five experimental groups (*n* = 10): Normal group, Model group, *c*POA groups intragastrically administered with 75 mg/kg/mouse (C-L group), 150 mg/kg/mouse (C-M group), or 300 mg/kg/mouse (C-H group). The mice received treatment for 4 weeks.

In the second experiment, following one-week acclimation period, 10 db/m mice were maintained on a basal diet, while 30 db/db mice were transitioned to a HFD for the experimental period. The body weight of each mouse was measured and the mice were allocated to the following experimental groups (*n* = 10): Control group, HFD group, *c*POA groups intragastrically administered with 150 mg/kg/mouse (C-150 group) or 300 mg/kg/mouse (C-300 group). This study used HFD-fed db/db mice to simulate the combined effects of severe metabolic syndrome in humans (obesity + diabetes + dietary imbalance), investigate the regulatory resilience of *c*POA in DAG shunting under extreme metabolic stress.

*c*POA was dissolved in a 1% sodium carboxymethylcellulose solution and homogenized to form a suspension. This suspension was administered daily at 9:00 AM via oral gavage for 4 weeks. The model group and the control group received an equivalent volume of the 1% sodium carboxymethylcellulose solution. The HFD comprised 10% lard, 10% sugar, 5% protein, 0.5% cholesterol, and 74.5% base feed by mass. The basal diet and HFD were purchased from Changzhou Cavens Laboratory Animal Co., Ltd. (Changzhou, China).

### 2.3. Reagents

*c*POA (CAS: 373-49-9) was prepared from sea buckthorn fruit oils via column chromatography and preparative high-performance liquid chromatography (prep-HPLC, Agilent, USA) [15]. Its purity (≥98%) was confirmed by Agilent 1260 HPLC and Agilent 7890A gas chromatography with flame ionization detection (GC-FID, AgilentTechnologies, Santa Clara, CA, USA) [16]. The chromatographic grade solvents methanol, acetonitrile, and methyl tert-butyl ether were obtained from Merck (Merck, Darmstadt, Germany). Formic acid and ammonium formate were sourced from ROE (ROE, Newark, DE, USA). The internal standards N-Heptadecanoyl-D-erythro-Sphingosylphosphorylcholine (SM(17:0), ≥99%), N-Heptadecanoyl-D-erythro-Sphingosine (Cer(17:0), ≥99%), 1,2-Diheptadecanoyl-sn-Glycero-3-Phosphoethanolamine (PE(17:0–17:0), ≥99%), and 1-heptadecanoyl-sn-glycero-3-phosphocholine (LPC(17:0), ≥99%) were all purchased from NU-CHEK (NU-CHEK, Elysian, MN, USA).

### 2.4. Sample Collection

The mice were fasted for 12 h before the final intragastric administration. Then, 1 h later, they were anesthetized by 40–80 mg/kg phenobarbital. The blood drawn from the retro-orbital sinus of every mouse was put into a centrifuge tube without anticoagulant. The blood samples were placed at room temperature for 30–60 min and separated by centrifugation at 4 °C at 3000× *g* for 10 min. The right lobe of the liver was snap-frozen in liquid nitrogen immediately after dissection, and the left lobe of the liver was immersed in paraformaldehyde for 48 h to perform HE and oil red staining for the pathological analysis. Serum and liver samples were stored at −80 °C until further analysis.

### 2.5. Extraction of Liver Metabolites

Added 20 mg of tissue to 200 μL of ultrapure water and homogenized for 5 min using a high-speed homogenizer (Eppendorf, Hamburg, Germany). Then, 20 μL of the homogenate was mixed with 225 μL of methanol solution and vortexed for 10 s. Next, 750 μL of methyl tert-butyl ether (MTBE) was added and vortexed for another 10 s. The mixture was subsequently vortexed for 10 min at 4 °C. After that, 188 μL of ultrapure water was added and vortexed for 20 s. The mixture was then centrifuged at 18,000 r/min for 2 min at 4 °C. 350 μL of the supernatant was transferred to a 1.5 mL centrifuge tube and dried at 45 °C for 2 h using a rotary evaporator. Next, 110 μL of methanol/toluene (9:1) mixed solution was added and vortexed for 10 min, followed by 10 min of centrifugation at 18,000 r/min. Finally, 90 μL of the supernatant was collected as the sample for further testing.

### 2.6. Lipidomics Analysis Method

The chromatographic analysis was performed using an Acquity UPLC BEH C18 column (2.1 × 100 mm, 1.7 μm) installed on an UltiMate 3000 RS UPLC system (Thermo Fisher Scientific, Sunnyvale, CA, USA), coupled to a Q-Exactive quadrupole-Orbitrap high-resolution mass spectrometer (Thermo Fisher Scientific, USA). The mobile phase consisted of two solutions: solution A containing acetonitrile and an aqueous solution (*v/v* = 40:60) of formic acid (0.1%) and formate amine (5 mM), and solution B containing isopropanol and acetonitrile (*v/v* = 9:1) with the same additives as solution A. The elution program was as follows: 0–4 min, 15% B; 4–5 min, 15–30% B; 5–22 min, 30–48% B; 22–23 min, 48–82% B; 23–30 min, 82–99% B. The flow rate was set to 0.30 mL/min, and the column temperature was maintained at 65 °C. Samples (1 μL) were injected into the system for analysis.

The mass spectrometry was performed in positive ion mode using a heating electrospray ion source (HESI) with a spray voltage of 3.5 kV. The ion source temperature was set to 306 °C and the capillary temperature was set to 300 °C. The sheath gas and auxiliary gas used were both nitrogen, with a sheath airflow of 275 kPa and an auxiliary airflow of 104 kPa. The mass-to-charge ratio (*m*/*z*) scanning range was set from 150 to 1800. LC-MS raw data files were converted to ABF file by ABF converter (https://www.reifycs.com/abfconverter/). MS-DIAL was used for data processing and lipid identification as described in our previous study [17]. ChemRICH was used for the chemical similarity analysis [18]. BioPAN was used for the lipidome metabolic pathway analysis [19]. MetaboAnalyst was used for the Partial Least Squares Discriminant Analysis (PLSDA) analysis.

### 2.7. RNA Extraction and Real-Time PCR Method

The protocols were adapted from established methodologies [20]. Hepatic tissues were homogenized in Trizol reagent (Thermo Fisher Scientific, USA) for total RNA isolation following the supplier’s guidelines. RNA purity and quantity were assessed via spectrophotometry (Nanodrop 2000c, Thermo Fisher Scientific), with samples exhibiting A260/A280 ratios between 1.8 and 2.0 selected for downstream processing. Reverse transcription was conducted with 1 μg RNA using Hifair™ 5× Strand cDNA Synthesis SuperMix (Yeasen, Shanghai, China).

SYBR Green-based amplification (Thermo Fisher Scientific) was performed on an LC480 thermocycler (Roche Diagnostics, Penzberg, Germany) with gene-specific primers (Generay Biotech, Shanghai, China; sequences listed in Table 1). Target gene expression was quantified via standard curve analysis and normalized against endogenous controls (β-actin and Gapdh). Triplicate technical and biological replicates were included in all assays.

### 2.8. Protein Extraction and Western Blot

The protocols were performed according to established methodologies [21]. Tissue homogenization was conducted in lysis buffer (Yeasen, China) supplemented with protease inhibitor cocktail (Qiagen, Hilden, Germany). Protein quantification was performed via BCA assay (Thermo Fisher Scientific), followed by SDS-PAGE resolution of lysates under reducing conditions. Electrophoretically resolved proteins were transferred onto PVDF membranes (Bio-Rad, Hercules, CA, USA) using semi-dry blotting systems.

Membranes were probed with β-actin primary antibody (Cell Signaling Technology, Danvers, MA, USA;#3700S; 1:2000 dilution), SREBP-1c (Abcam, Cambridge, UK; ab28481; 1:2000 dilution), SCD-1 (Cell Signaling Technology, Danvers, MA, USA; #2794S; 1:2000 dilution) and species-matched HRP-conjugated secondary antibodies (1:2000). Chemiluminescent detection was achieved using Western HRP substrate (Cell Signaling Technology, Danvers, MA, USA). Signal acquisition and analysis were performed in triplicate biological replicates.

### 2.9. Statistical Analysis

All the experimental data visualized by bar plots were presented as means ± standard deviations. Statistical significance was analyzed using one-way ANOVA and Tukey’s post hoc test, as appropriate, in GraphPad Prism 8. Statistical significance was defined as * *p* < 0.05, ** *p* < 0.01, and *** *p* < 0.001.

## 3. Results and Discussion

### 3.1. cPOA and Obesity Mouse with Basal Feed

#### 3.1.1. Mouse Body Weight and Fat Index

**Figure 1:**
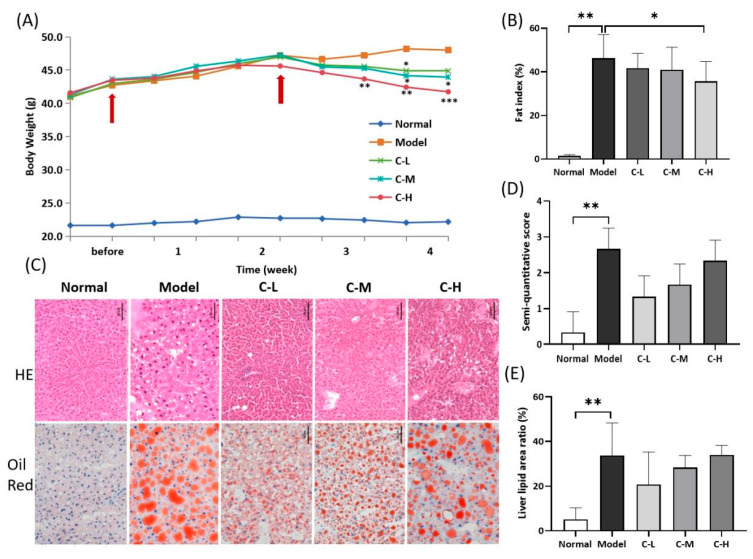
Physiological and pathological indicators: (**A**) Body weight (*n* = 10), (**B**) Fat index (*n* = 10), (**C**) Schematic diagram of liver pathological sections (HE and Oil Red) (*n* = 3), (**D**,**E**) Statistical analysis of liver histopathological results (*n* = 3). * *p* < 0.05, ** *p* < 0.01, *** *p* < 0.001.

Body weight was measured twice weekly, and the fat index was calculated at the end of the study. The body weight in the model group increased steadily throughout the research period (Figure 1A). In the *c*POA groups, a decreasing trend in body weight was observed starting from the second week, with the C-H group showing a significant reduction (*p* = 0.0194). The C-L and C-M groups also exhibited relative weight loss, achieving statistical significance by the fourth week (*p* = 0.0391 and *p* = 0.0316, respectively), while the C-H group demonstrated an even more reduction (*p* = 0.0010). This indicated that *c*POA reduced the body weight of obese mice in a dose-dependent manner.

Obesity significantly increased the physiological burden on internal organs [22]. The expansion of both subcutaneous and visceral white adipose tissue led to fat accumulation: a thick layer encased the organ surfaces, while lipid content also rose within the parenchyma [23]. This increase in fat deposition further elevated functional demand on the organs, resulting in compensatory enlargement or hypertrophy [24]. After *c*POA administration, the fat index exhibited a declining trend with a dose-dependent effect. The C-H group showed a significant reduction in fat index compared to the model group (*p* = 0.0386) (Figure 1B).

#### 3.1.2. Hepatic Pathological Analysis

Oil Red O staining of the normal group revealed minimal red lipid droplets in liver tissues, indicating negligible fat accumulation. Correspondingly, H&E staining demonstrated intact hepatic architecture with minimal vacuolation and only small lipid droplets. In contrast, the model group exhibited abundant, intensely stained large lipid droplets via Oil Red O, filling most hepatocytes. H&E staining confirmed pronounced steatosis, showing severe vacuolation, nuclear pyknosis, and necrosis. The C-L group displayed reduced lipid droplets with Oil Red O staining compared to the model group, although mild steatosis persisted. The H&E staining group revealed decreased vacuolation, localized inflammatory cell infiltration, and smaller residual lipid droplets. Oil Red O staining of the C-M group showed prominent lipid droplets, corresponding to extensive fatty lesions. H&E staining highlighted heterogeneous vacuole sizes, nuclear pyknosis, and partial hepatocyte lysis. The C-H group exhibited large lipid droplets and severe hepatic injury. H&E staining showing confirmed extensive cellular dissolution and vacuolation (Figure 1C).

Using ImageJ2 software, lipid droplet area (Oil Red O) and vacuolation (H&E) were quantified as the ratio of total lipid droplet area to tissue area (Figure 1D,E). The results demonstrated that lower doses of *c*POA reduced hepatic lipid accumulation. However, higher *c*POA doses correlated with an increasing trend in lipid droplet area, inconsistent with the fat index. This suggested that elevated lipid droplets observed at higher doses may arise from transient hepatic storage of *c*POA or its metabolites.

#### 3.1.3. Analysis of Liver Lipidome

Liver is the strongest organ with the lipogenic ability in the body, which synthesizes fat in the cytoplasm. Therefore, lipidomics was used to analyze the metabolic pathway of *c*POA following its entry into the body, thereby elucidating its potential mechanisms of weight and fat reduction.

##### PLSDA

**Figure 2:**
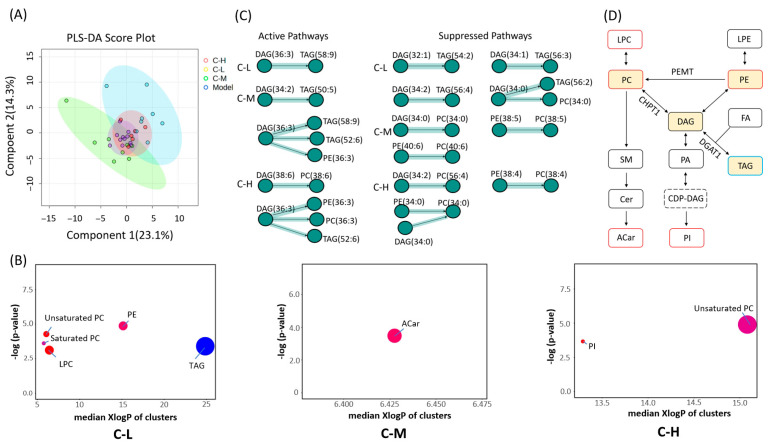
Lipidomic analysis of liver tissues from each group with basal feed (*n* = 10): (**A**) PLSDA score plot of liver tissue samples, (**B**) ChemRICH analyze: Clusters were generated by chemical similarity and ontology mapping. The plot y-axis showed the most significantly altered clusters on the top. Cluster colors give the proportion of increased or decreased compounds (red = increased, blue = decreased), (**C**) Pathway analysis of lipid species: The nodes correspond to lipids and the directed edges between two nodes symbolize a reaction between these two lipids, the nodes shape denoted the class of lipids, (**D**) Diagram of DAG metabolic shunting and transformation (red = increased, blue = decreased).

The disease model group and the *c*POA groups gradually separated on the score plot (Figure 2A), indicating significant differences in lipidomic profiles. The varying degrees of separation observed with different dose groups suggested the presence of a dose effect.

##### Chemical Similarity Enrichment Analysis

The results showed that TAG was significantly decreased at C-L group, which was consistent with the pathological Oil Red O staining results in the liver. While, LPC, unsaturated PC, saturated PC, PE were significantly increased. ACar increased at C-M group, and unsaturated PC and PI were significantly increased in C-H group (Figure 2B). The results showed that low-dose *c*POA preferentially reduced hepatic TAG accumulation. As the *c*POA dose increased, however, the metabolism of ACar and PC/PE became predominant in the liver. This shift may be associated with dose-dependent differences in enzymatic activity or the regulation of metabolic branch points [25,26]. ACar, an important intermediate in fatty acid β-oxidation, likely facilitated enhanced fatty acid β-oxidation with increasing *c*POA doses, consisted with the significant dose-dependent reduction in the fat index.

##### Pathway Analysis of Lipid Species

In this analysis, we explored systematic changes in lipid pathways at the species level (Figure 2C and Figure S1). The results showed that all three *c*POA dose groups activated the metabolic conversion pathway of DAG(36:3), although the downstream metabolites of DAG(36:3) conversion varied across dose groups. In the C-L group, the signaling pathway of DAG(36:3) conversion to TAG(58:9) was activated. The C-M group activated pathways for DAG(36:3) conversion to PE(36:3), TAG(52:6), and TAG(58:9), suggesting that the metabolic branching of DAG(36:3) extended to PE(36:3) and other TAG subtypes (e.g., TAG(52:6)), with partial metabolic resources potentially allocated to membrane phospholipid synthesis. In contrast, the C-H group promoted DAG(36:3) conversion to PC(36:3), PE(36:3), and TAG(52:6), demonstrating a further shift in metabolic pathways toward the production of PC(36:3) and PE(36:3), while retaining partial TAG(52:6) synthesis. This implies that high-dose *c*POA preferentially supports the generation of structural phospholipids rather than mere lipid storage.

Other DAG also underwent activation or inhibition. For example, DAG(34:0) was inhibited from converting to PC(34:0) at all dose-groups, and at C-L group, it was also inhibited from converting to TAG(56:2). DAG(34:2) was inhibited from converting to TAG(56:4) at C-L and C-H groups, while DAG(38:6) was activated to convert to PC(38:6) at the C-H group (Figure 2C).

Results from the basal diet experiment indicated that oral gavage of *c*POA modulated the metabolic interconversion of TAG, PC, and PE through a dose-dependent DAG metabolic shunt. This primarily involved three distinct interconversion pathways: the activation or inhibition of DAG→TAG, DAG→PC, and suppression of PE→PC (Figure 2D). Interestingly, DAG (36:3) was activated to generate two TAG subtypes, which contradicted the relatively low content of TAG in the liver.

##### Lipid Reactions and Differential Lipids

**Figure 3:**
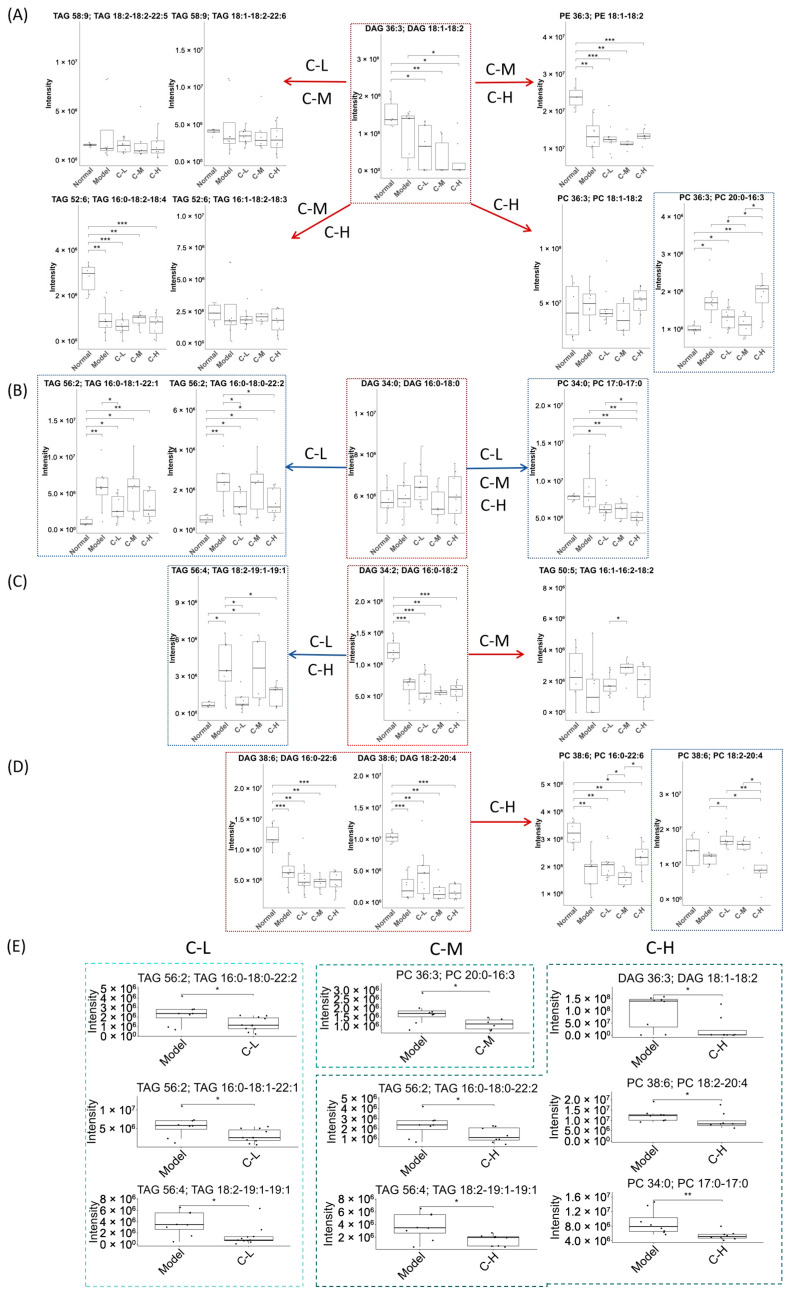
Lipid reactions and differential metabolites (*n* = 10): (**A**–**D**) Lipid reactions: the arrows, red = activated, blue = suppressed, the compounds in the red box were DAGs, and the compounds in the blue box were metabolites with significant changes, (**E**) Differential metabolites of each group. * *p* < 0.05, ** *p* < 0.01, *** *p* < 0.001.

During the metabolic shunting pathway of DAG, several DAG species, including DAG(36:3, 18:1–18:2), DAG(34:0, 17:0–17:0), and DAG(34:2, 16:0–18:2), exhibited pronounced substrate preference, with their metabolic flux predominating in this branching pathway (Figure 3A–D). Specifically, *c*POA dynamically regulated the metabolic fate of DAG(36:3, 18:1–18:2) through a dose-dependent mechanism, shifting from lipid storage at lower doses to phospholipid synthesis at higher doses (Figure 3A). In contrast, other species such as DAG(38:6, 18:2–20:4) and DAG(38:6, 16:0–22:6) were specifically activated to convert to corresponding PC in the C-H group (Figure 3D).

Among the experimental groups, the C-L group showed significant reductions in TAGs, specifically TAG(56:2, 16:0–18:0–22:2), TAG(56:2, 16:0–18:1–22:1), and TAG(56:4, 18:2–19:1–19:1) (*p* < 0.05). In contrast, the C-M group exhibited a marked decrease in PC(36:3, 20:0–16:3) (*p* < 0.05). Notably, the C-H group demonstrated the most pronounced reductions, affecting DAG(36:3, 18:1–18:2), PC(38:6, 18:2–20:4), PC(34:0, 17:0–17:0), and TAG species TAG(56:2, 16:0–18:0–22:2) and TAG(56:4, 18:2–19:1–19:1) (*p* < 0.05; Figure 3E).

Abnormal accumulation of TAG and DAG was a hallmark of metabolic diseases such as MASLD and obesity, with subtype-specific variations reflecting lipid metabolic imbalances at different pathological stages [27].

Notably, hepatic sn-1,2-diacylglycerol (sn-1,2-DAG) has been identified as a key contributor to obesity-related lipid disorders in both rodent and human studies, driving hepatic insulin resistance and type 2 diabetes [28]. Specific DAG subtypes—including DAG(18:1/18:1) and DAG(16:0/18:1)—promoted inflammatory and oxidative stress responses by activating protein kinase C (PKC) pathways, thereby exacerbating cardiovascular risks [29]. TAG subtypes such as TAG(56:2, 16:0–18:0–22:2) and TAG(56:4, 18:2–19:1–19:1) impacted cardiovascular health by regulating inflammatory pathways, aggravating hepatic lipid accumulation, and inducing insulin resistance [30].

PC subtypes containing long-chain saturated fatty acids, such as PC(36:3, 20:0–16:3), were associated with vascular endothelial dysfunction through modulation of membrane fluidity and thrombus formation [31]. PC subtypes with polyunsaturated fatty acids (PUFA) generally exhibited inverse associations with cardiovascular disease risk [32]. However, studies indicated that highly unsaturated PC species rich in arachidonic acid (20:4)—exemplified by PC(38:6, 18:2–20:4)—might exacerbate atherosclerosis via pro-inflammatory signaling. Additionally, saturated PC subtypes (e.g., PC(34:0, 17:0–17:0)) contributed to hepatic PC/PE ratio imbalance, promoting insulin resistance [33].

Consequently, The metabolic shunting strategy applied here operates on a “Robin Hood” principle—redirecting resources from harmful pathways toward beneficial ones. Within this analogy, the DAG pool represents communal wealth, comprising both lipotoxic DAG species and physiologically essential DAGs that maintain metabolic homeostasis. Acting as the metabolic “Robin Hood”, *c*POA at varying doses regulated the metabolic shunting of specific DAG species (e.g., DAG(36:3, 18:1–18:2)), reduced the relative levels of TAGs (e.g., TAG(56:2, 16:0–18:0–22:2)) and PCs (e.g., PC(38:6, 18:2–20:4)), and thereby altered lipid storage, lipid oxidation, and cell membrane fluidity/flexibility, regulated lipid metabolism homeostasis.

### 3.2. cPOA and Obesity Mouse with HFD

#### 3.2.1. Mouse Body Weight and Fat Index

**Figure 4:**
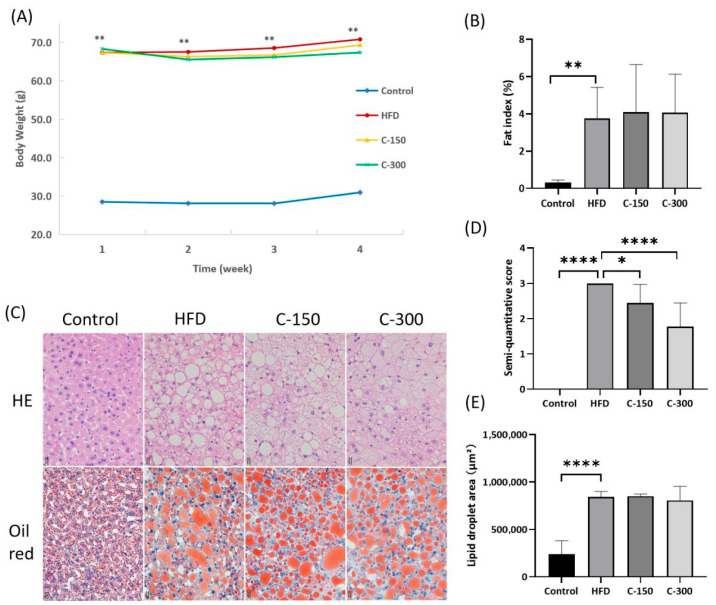
Physiological and pathological indicators: (**A**) Body weight (*n* = 10), (**B**) Fat index (*n* = 10), (**C**) Schematic diagram of liver pathological sections (HE and Oil Red) (*n* = 3), (**D**,**E**) Statistical analysis of liver histopathological results (*n* = 3). * *p* < 0.05, ** *p* < 0.01, **** *p* < 0.0001.

The disease model group exhibited significant increases in both body weight (*p* < 0.0001) and epididymal fat index (*p* < 0.01) due to HFD feeding. However, *c*POA administration failed to reduce body weight significantly, and the fat index remained unchanged (Figure 4A,B). This suggested that the HFD-induced systemic lipid metabolism disorder may have masked the localized effects of *c*POA, possibly due to excessive metabolic overload.

#### 3.2.2. Hepatic Pathological Analysis

Histological assessment revealed distinct hepatic alterations across experimental groups (Figure 4C). Control mice exhibited well-defined lobular architecture with irregularly radiating hepatocyte cords, without notable lipid accumulation or vascular congestion. In contrast, HFD-fed mice displayed obscured lobular boundaries, disorganized hepatocyte cords, narrowed hepatic sinusoids, and hepatocyte enlargement with cytoplasmic lipid vacuoles and pallor, as well as pronounced sinusoidal congestion and inflammatory infiltration. Administration of 150 mg/kg *c*POA (C-150) partially restored lobular integrity compared to the HFD group (*p* < 0.05; Figure 4D). The 300 mg/kg *c*POA group (C-300) demonstrated further improvements, including relatively defined lobular organization, regularized cord alignment, and attenuated inflammatory or congestive manifestations (*p* < 0.0001 vs. HFD). Notably, hepatic lipid droplet content of *c*POA groups remained unchanged (Figure 4E). These findings indicate that *c*POA dose-dependently mitigates diet-induced hepatic pathology, with higher doses enhancing structural restoration and inflammatory resolution.

#### 3.2.3. Analysis of Liver Lipidome

##### PLSDA

**Figure 5:**
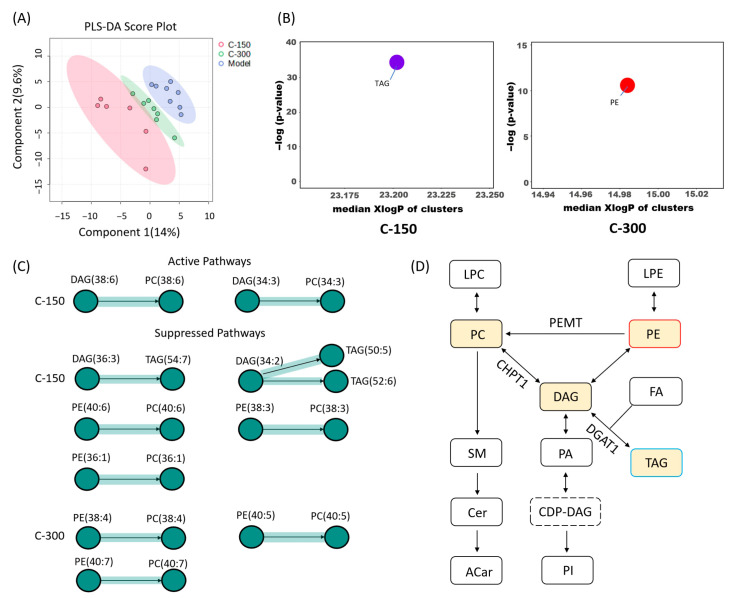
Lipidomic analysis of liver tissues of HFD-fed mouse groups (*n* = 10): (**A**) PLSDA score of liver tissue samples, (**B**) ChemRICH analysis: Clusters were generated by chemical similarity and ontology mapping. The plot y-axis showed the most significantly altered clusters on the top. Cluster colors give the proportion of increased or decreased compounds (red = increased, blue = decreased), (**C**) Pathway analysis of lipid species: The nodes correspond to lipids and the directed edges between two nodes symbolize a reaction between these two lipids, the nodes shape denoted the class of lipids, (**D**) Diagram of metabolic shunting mechanism of DAG (red = increased, blue = decreased).

Principal component analysis revealed progressive segregation between the HFD group and *c*POA-treated groups on the score plot (Figure 5A), indicating significant intergroup metabolic profile differences. The dose-dependent spatial separation patterns further suggested a graded effect of *c*POA administration.

##### Chemical Similarity Enrichment Analysis

Enrichment analysis revealed differential alterations between *c*POA dose groups (Figure 5B): the 150 mg/kg dose group (C-150) showed significantly reduced TAG enrichment, whereas the 300 mg/kg group exhibited significantly elevated PE enrichment (C-300). These findings suggest that under HFD conditions, low-dose *c*POA maintained suppression of TAG biosynthesis, whereas high-dose *c*POA concurrently enhanced PE metabolism. This dose-dependent shift in lipid partitioning aligns with histopathological evidence of hepatic structural and functional improvements.

##### Pathway Analysis of Lipid Species

Under HFD feeding conditions, distinct dose-dependent modulations of lipid metabolic pathways were observed, particularly in DAG-related remodeling processes. In the 150 mg/kg dose group (C-150), the conversion of DAG(36:3), DAG(34:2) to TAG(54:7), TAG(50:5) and TAG(52:6) were significantly suppressed, Conversely, pathways mediating the transformation of DAG(34:3) and DAG(38:6) into PC(34:3) and PC(38:6), respectively, were markedly activated. Additionally, the conversion of PE(40:6) and PE(38:6) conversion to PC(40:6) and PC(38:6), as well as the PE(36:1)-to-PC(36:1) pathway, was inhibited (Figure 5C and Figure S2). In contrast, the higher dose (C-300) mainly and selectively disrupted PE-to-PC metabolism, specifically suppressing the conversion of PE(38:4), PE(40:5), and PE(40:7) into their corresponding PC species. These findings indicated dose-dependent alterations in lipid flux: intake of lower *c*POA doses reduced TAG accumulation primarily by remodeling the DAG-to-TAG pathway, whereas higher *c*POA doses shifted the metabolic equilibrium toward PC and PE homeostasis (Figure 5D), thereby alleviating HFD-exacerbated hepatic injury.

Integrated analysis of db/db mice fed either basal diet or HFD revealed that oral *c*POA administration initially attenuated hepatic TAG accumulation. As the *c*POA dose increased, it further activated PC/PE metabolism, mitigating hepatic damage exacerbated by HFD. In basal-diet mice, the hepatic injury primarily stemmed from the genetic defect (Lepr^−/−^), *c*POA likely alleviated lipid accumulation by activating long-chain fatty acids into ACar, thereby enhancing fatty acid β-oxidation. In contrast, under HFD conditions, β-oxidation was inhibited, diverting ACar preferentially toward lipogenesis [34]. This shift potentially explains the unchanged adiposity coefficient and suggests that HFD-induced metabolic stress may attenuate the efficacy of *c*POA.

##### Lipid Reactions and Differential Metabolites

**Figure 6:**
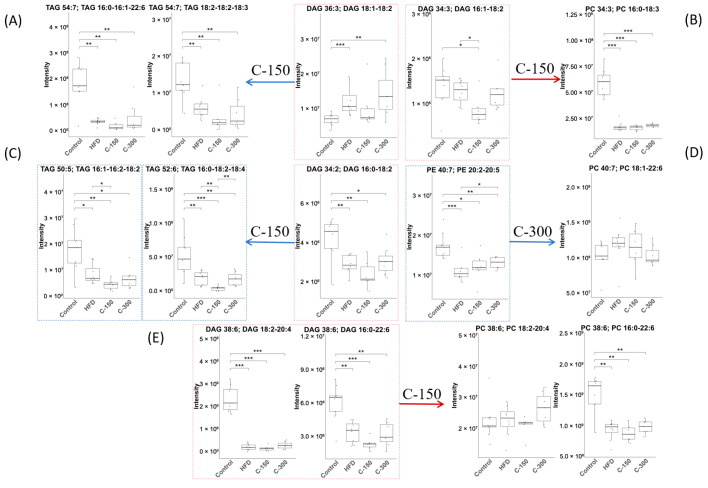
Lipid reactions and differential metabolites (*n* = 10): The red arrows represent the activating response, and the blue arrows represent the inhibitory response; the compounds in the red box were the DAGs, and the compounds in the blue box were metabolites with significant changes. (**A**) DAG(36:3)→TAG(54:7) inhibited in C-150 group; (**B**) DAG(34:3)→PC(34:3) activated in C-150 group; (**C**) DAG(34:2)→TAG(50:5) & TAG(52:6) inhibited in C-150 group; (**D**) PE(40:7)→PC(40:7) inhibited in C-300 group; (**E**) DAG(38:6)→PC(38:6) activated in C-300 group. * *p* < 0.05, ** *p* < 0.01, *** *p* < 0.001.

Under HFD conditions, the model group exhibited an extremely significant increase in DAG(36:3, 18:1–18:2) levels compared to the normal diet group (*p* < 0.001) (Figure 3A and Figure 6A). This indicated that HFD markedly promoted DAG(36:3, 18:1–18:2) generation and exacerbated cardiovascular and cerebrovascular diseases risk [35]. These findings further suggested that DAG(36:3, 18:1–18:2) might be a direct intrinsic factor in HFD-induced aggravation of lipid metabolism disorders. In the 150 mg/kg dose group (C-150), DAG(36:3, 18:1–18:2) showed a decreasing trend. However no significant change occurred in the 300 mg/kg group (C-300). Additionally, metabolic conversion of DAG(36:3, 18:1–18:2) to TAG(54:7, 16:0–16:1–22:6) and TAG(54:7, 18:2–18:2–18:3) displayed a non-significant reduction trend (Figure 6A).

Concurrently, DAG(34:3, 16:1–18:2) and DAG(38:6, 18:2–20:4) showed activated conversion to PC (Figure 6B,E). Notably, in the C-150 group, DAG(34:3, 16:1–18:2) level was significantly reduced (*p* < 0.05). Under both basal feed and HFD conditions, DAG(34:2, 16:0–18:2) underwent conversion to TAG with significant differences (*p* < 0.05, Figure 6C), though the resulting TAG subtypes differed between groups. Furthermore, *c*POA induced significant metabolic alterations in DAG(34:3, 16:1–18:2), TAG(50:5, 16:1–16:2–18:2), TAG(52:6, 16:0–18:2–18:4), and PE(40:7, 20:2–20:5) (Figure 6A–E). The results demonstrated that *c*POA reduced DAG-to-TAG conversion and inhibited PE-to-PC remodeling under HFD conditions.

In addition, the *c*POA intervention group showed increased unsaturation of fatty acid chains during DAG or PE conversion, which potentially alleviated liver inflammation [36]. Mechanistically, excessive hepatic lipid accumulation combined with inadequate mitochondrial adaptation promoted DAG and ceramide generation. Critically, specific DAG subtypes (e.g., C18:1-DAG and sn-1,2-DAG) localized to the plasma membrane activated novel protein kinase Cε (nPKCε), thereby driving insulin resistance and MASLD progression [37].

DAG regulated hepatic and skeletal muscle expression of lipases and lipid metabolism genes, inhibited fatty acid synthase (FASN) activity, while enhanced fatty acid oxidation enzymes. This coordinated action upregulated lipid β-oxidation while suppressing hepatic gluconeogenesis and stimulating muscle fatty acid oxidation [38,39]. In non-alcoholic steatohepatitis (MASH) patients, plasma DAG levels were significantly elevated. Specifically, subtypes including DAG(36:3, 18:1–18:2), DAG(36:2, 18:1–18:1), DAG(36:1, 18:0–18:1), and DAG(34:1, 16:1–18:0) showed marked increases versus controls [35]. Notably, elevated DAG(36:3, 18:1–18:2) was strongly associated with lipotoxicity [40], a key driver that induced hepatocyte injury, inflammation, insulin resistance, and liver disease exacerbation [41].

Certain PC subtypes also exhibited altered levels in MASH patients. Specifically, PC(32:1, 16:0–16:1) and PC(36:1, 18:0–18:1) levels were significantly elevated, whereas PC(40:5, 18:0–22:5) concentrations decreased [42]. As critical membrane components, these PCs maintained hepatocyte membrane stability and fluidity. Alterations in PC levels compromised cellular function and metabolism, further disrupting lipoprotein formation and lipid transport [43]. Similarly, TAG—the primary energy storage form—showed direct links to obesity-related diseases when abnormally elevated. Elevated TAG induced hepatocyte damage and inflammation [44], while also impairing lipoprotein metabolism and overall lipid homeostasis [41].

Integrated analyses of normal diet and HFD experiments demonstrated that oral *c*POA administration induced dose-dependent metabolic remodeling by regulating DAG shunting, where HFD significantly elevated DG(36:3,18:1–18:2) levels as a potential biomarker for hepatic injury. Specifically, cPOA treatment inhibited DAG-to-TAG conversion in low-dose groups while activating DAG-to-PC conversion at medium doses and suppressing PE-to-PC conversion in medium/high-dose groups. Notably, key metabolic convergence points across cohorts included DAG(36:3,18:1–18:2), DAG(38:6,18:2–20:4), DAG(38:6,16:0–22:6), and DAG(34:2,16:0–18:2) with key reactions: DAG(34:2)→TAG(50:5), DAG(38:6)→PC(38:6), PE(38:4)→PC(38:4), PE(40:6)→PC(40:6). These changes collectively indicated that *c*POA intervention modulated PC/PE metabolism, reduced TAG synthesis, and altered fatty acid oxidation, ultimately attenuating body weight gain and exerting hepatoprotection.

### 3.3. Genetic- and Protein-Level Verification

To validate these metabolic pathway findings, key pathway proteins were analyzed via qPCR and Western blot assays.

#### 3.3.1. *c*POA Regulation of DAG Metabolic Branching Enzymes

**Figure 7:**
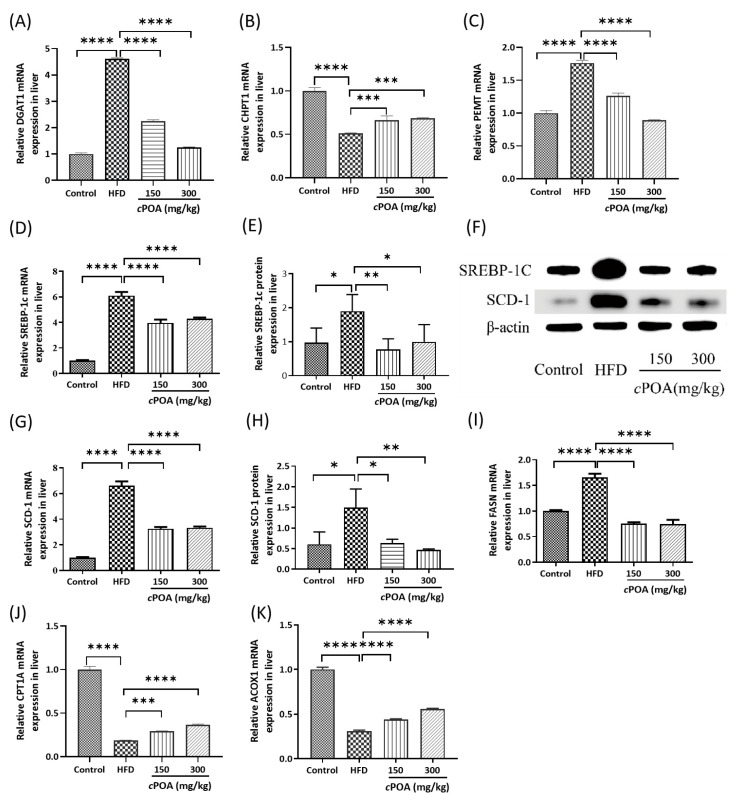
qPCR and Western blot for gene and protein expression: (**A**) Relative content of DGAT1genes, (**B**) Relative content of CHPT1 genes, (**C**) Relative content of PEMT genes, (**D**) Relative content of SREBP-1c genes, (**E**) Relative content of SREBP-1c proteins, (**F**) Western blot of SREBP-1c and SCD-1 proteins, (**G**) Relative content of SCD-1genes, (**H**) Relative content of SCD-1 proteins, (**I**) Relative content of FASN genes, (**J**) Relative content of CPT1A genes, and (**K**) Relative content of ACOX1 proteins, * *p* < 0.05, ** *p* < 0.01, *** *p* < 0.001, **** *p* < 0.0001, *n* = 3.

DGAT1 (diacylglycerol acyltransferase 1), the key enzyme catalyzing the final step of TAG synthesis, was directly associated with insulin resistance, enhanced lipogenesis, and fatty liver development in diabetic/obese models. Although hepatic DGAT1 esterified exogenous fatty acids into triglycerides for lipid droplet storage, which provided lipotoxicity protection, its pathological overexpression in MASLD promoted lipid accumulation. This overload subsequently induced endoplasmic reticulum (ER) stress, oxidative stress, hepatic inflammation, and fibrosis [45]. Conversely, DGAT1 inhibition alleviated these stresses and mitigated MASLD progression [46]. Consistently, qPCR revealed extremely significant DGAT1 upregulation (*p* < 0.0001) in HFD group, positively correlating with hepatic TAG content, whereas *c*POA intervention caused profound downregulation (*p* < 0.0001) (Figure 7A). This transcriptional regulation was likely mediated by SREBP-1c, the master upstream lipogenic regulator [47].

Lipid droplets (LDs) served as intracellular organelles storing TAGs, with their surface monolayer phospholipid structure directly linked to PC synthesis [48]. Under HFD conditions, hepatic lipid accumulation and oxidative stress were exacerbated, inducing lipotoxicity [49]. Concurrently, choline phosphotransferase 1 (CHPT1), the Kennedy pathway enzyme catalyzing DAG-to-PC conversion, showed downregulated expression. This impaired PC synthesis, disrupted membrane phospholipid composition, and destabilized LD-coating proteins like perilipin 2 (PLIN2), thereby triggering LD fusion and hepatocyte injury [48]. Notably, enhanced PC synthesis optimized membrane integrity and LD stability, establishing a protective feedback loop. As demonstrated (Figure 7B), HFD significantly suppressed hepatic CHPT1 (*p* < 0.0001) by inhibiting insulin signaling and elevating oxidative stress, resulting in PC synthesis dysfunction and lipotoxicity. In contrast, *c*POA gavage likely mitigated hepatic lipid metabolic disorders by activating PPARα, upregulating CHPT1 expression, and facilitating the conversion of DAG species (e.g., DAG(38:6) and DAG(34:3)) to PC.

Phosphatidylethanolamine N-methyltransferase (PEMT) catalyzed PE methylation to generate PC, providing an alternative PC synthesis pathway [50]. Critically, the PC/PE molar ratio maintained membrane integrity, whereas reduced PC or excess PE disrupted mitochondrial and ER membrane dysfunction, and exacerbate oxidative stress and ER stress [11]. In db/db mice, insulin resistance and HFD-induced choline deficiency significantly upregulated PEMT transcription (*p* < 0.0001), thereby activated the PEMT pathway to sustain PC supply, supported VLDL assembly, and promoted lipid efflux [51]. Following oral administration of *c*POA, a marked downregulation of PEMT transcription (*p* < 0.0001) was observed, accompanied by inhibition of PE-to-PC conversion (Figure 7C). The study speculated that *c*POA indirectly suppressed PEMT via SREBP-1c or other lipogenic transcription factors. Notably, *c*POA-mediated PEMT inhibition (PE→PC) concurrently activated the CDP-choline pathway, thereby increasing hepatic dependence on CHPT1. To prevent lipotoxicity, the liver likely maintained CHPT1 expression through feedback mechanisms, ensuring timely conversion of DAG to PC [50].

Changes in DGAT1, CHPT1, and PEMT gene expression substantiated that *c*POA dynamically modulated hepatic lipid metabolism via a dose-responsive DAG metabolic shunting mechanism, which reduced lipid accumulation and alleviated liver injury.

#### 3.3.2. Fat Synthesis Related Enzyme Genes and Protein Expression

Fat synthesis was a tightly regulated metabolic process involving key enzymes and transcription factors. Sterol regulatory element-binding protein 1c (SREBP-1c) acted as a master transcriptional regulator, driving the expression of ACC, FASN, and SCD-1 in response to insulin and nutrient availability [52]. Acetyl-CoA carboxylase (ACC) catalyzed the carboxylation of acetyl-CoA to malonyl-CoA, which was the rate-limiting step in fatty acid synthesis. Fatty acid synthase (FASN) then elongated and saturated malonyl-CoA into palmitate, the primary long-chain fatty acid. Stearoyl-CoA desaturase 1 (SCD-1) further introduced double bonds to generate unsaturated fatty acids [53]. Additionally, Dysregulation of these enzymes in liver or adipose tissue was closely linked to lipid accumulation, insulin resistance, and metabolic disorders, such as obesity and MASLD. qPCR and Western blot analyses demonstrated that in the HFD group, the expression levels of SREBP-1c, SCD-1 and FASN genes were significantly elevated, accompanied by increased body weight and fat mass. In contrast, the *c*POA-treated groups exhibited marked reductions in both gene and protein expression of SREBP-1c, SCD-1 and FASN gene (Figure 7D–I), and thereby suppressed fatty acid de novo synthesis.

#### 3.3.3. Gene and Protein Expression of β-Oxidation Related Enzymes

*c*POA was a natural ligand of PPARα, which primarily regulated fatty acid β-oxidation and lipid catabolism. Upon activation, PPARα promoted fatty acid β-oxidation in the liver and muscle, inhibited fat accumulation, and modulated the expression of oxidation-related key enzymes, including Carnitine Palmitoyltransferase 1A (CPT1A) and Acyl-CoA Oxidase 1 (ACOX1) [54]. Consequently, qPCR results revealed that the expression of ACOX1 and CPT1A genes was significantly reduced in the HFD group (*p* < 0.01) (Figure 7J,K). By contrast, in the *c*POA-treated groups, the expression of these genes increased dramatically (*p* < 0.0001), suggesting that *c*POA enhanced fatty acid oxidation and reduced lipid accumulation.

## 4. Conclusions

The results of this study demonstrated that *c*POA dynamically regulated lipid metabolism through a dose-dependent DAG metabolic shunting mechanism. Low-dose *c*POA inhibited DAG-to-TAG synthesis, while higher doses redirected DAG into PC/PE biosynthetic pathways, thereby reducing hepatic lipid accumulation, decreasing body weight, and lowering adiposity index. Mechanistically, *c*POA downregulated lipogenic genes and proteins (SREBP-1c, SCD-1, FAS) and upregulating fatty acid β-oxidation enzymes (CPT1A, ACOX1), thereby modulating lipid homeostasis. Notably, DAG(36:3, 18:1–18:2) was identified as a potential biomarker for HFD-aggravated lipid metabolic disorders. Collectively, our findings establish *c*POA as a novel bidirectional regulator of lipid metabolism in mice, capable of simultaneously modulating both lipid synthesis and oxidation pathways. The dose-dependent metabolic effects and pathway-specific actions revealed in this study provide a foundation for developing precision interventions against obesity and related metabolic disorders. *c*POA (C16:1) is abundantly present in nature and can serve as a lipid supplement for obese individuals or as a food oil additive.

## Figures and Tables

**Table 1:** Primers used for real-time PCR.

Gene	Forward Primer	Reverse Primer
GAPDH	5′-ATCATCTCCGCCCCTTCTG-3′	5′-GTGATGGCATGGACTGTGG-3′
β-actin	5′-TATGCTCTCCCTCACGCCATCC-3′	5′-GTCACGCACGATTTCCCTCTCAG-3′
DGAT1	5′-CTCAACTTTCCTCGGTCCCC-3′	5′-GATCAGCCCCACTTGAAGCT-3′
CHPH1	5′-GGAGGAGCAACAATGTGGGA-3′	5′-ACCCATTCTTGCCAACACCA-3′
PEMT	5′-CCACTGCTTCACACAGGCTA-3′	5′-AACCTAGGAATGCAAGGCCC-3′
SREBP-1c	5′-GAGCGAGCGTTGAACTGTAT-3′	5′-ATGCTGGAGCTGACAGAGAA-3′
SCD-1	5′-TTCTTGCGATACACTCTGGTGC-3′	5′-CGGGATTGAATGTTCTTGTCGT-3′
FASN	5′-CAAGTGTCCACCAACAAGCG-3′	5′-GGAGCGCAGGATAGACTCAC-3′
ACOX1	5′-GGGTGCTGATGCTGTGGATGTC-3′	5′-GGGTGAGGTCCAACCAGAGAGG-3′

## Data Availability

The original contributions presented in the study are included in the article/Supplementary Materials, further inquiries can be directed to the corresponding author.

## Supplementary Materials

The following supporting information can be downloaded at: https://www.mdpi.com/article/10.3390/foods14142504/s1, Figure S1: Pathway analysis of lipid species under basal food conditions; Figure S2: Pathway analysis of lipid species under HFD conditions.

## Abbreviations

*c*POA	*Cis*-palmitoleic acid
LPC	Lysophosphatidylcholine
PC	Phosphatidylcholine
PE	Phosphatidylethanolamine
LPE	Lysophosphatidylethanolamine
DAG	Diacylglycerol
PA	Phosphatidic Acid
CDP-DG	CDP-diacylglycerol
ACar	Acylcarnitine
PI	Phosphatidylinositol
TAG	Triacylglycerol
FA	Fatty Acid
PEMT	Phosphatidylethanolamine N-methyltransferase
DGAT1	Diacylglycerol O-Acyltransferase 1
CHPT1	Choline phosphotransferase 1
SREBP-1c	Sterol regulatory element-binding protein 1c
SCD-1	Stearoyl-CoA desaturase 1
FAS	Fatty acid synthase
CPT1A	Carnitine Palmitoyltransferase 1A
ACOX1	Acyl-CoA Oxidase 1
