# Supplementary material for: Cis-Palmitoleic Acid Regulates Lipid Metabolism via Diacylglycerol Metabolic Shunting
Source: Foods. 2025 Jul 17;14(14):2504. doi: 10.3390/foods14142504 (PMC12294437; doi:10.3390/foods14142504)
Supplement: Supplementary file 1 [file foods-14-02504-s001.zip › foods-3733717-supplementary.pdf]

Figure S1. Pathway analysis of lipid species under basal food conditions: The nodes correspond to lipids and the directed edges between two nodes symbolise a reaction between these two lipids. The nodes shape denotes the class of lipids and their colour depends on the pathway status (active/suppressed or none). The colour of the edges depends on the value of the Z-score: green for positive scores and red for negative scores. The reactions with a particular status were highlighted.

Figure S2. Pathway analysis of lipid species under HFD conditions: The nodes correspond to lipids and the directed edges between two nodes symbolise a reaction between these two lipids. The nodes shape denotes the class of lipids and their colour depends on the pathway status (active/suppressed or none). The colour of the edges depends on the value of the Z-score: green for positive scores and red for negative scores. The reactions with a particular status were highlighted.
