# Supplementary figures and images for: Cis-Palmitoleic Acid Regulates Lipid Metabolism via Diacylglycerol Metabolic Shunting
Source: Foods. 2025 Jul 17;14(14):2504. doi: 10.3390/foods14142504 (PMC12294437; doi:10.3390/foods14142504)

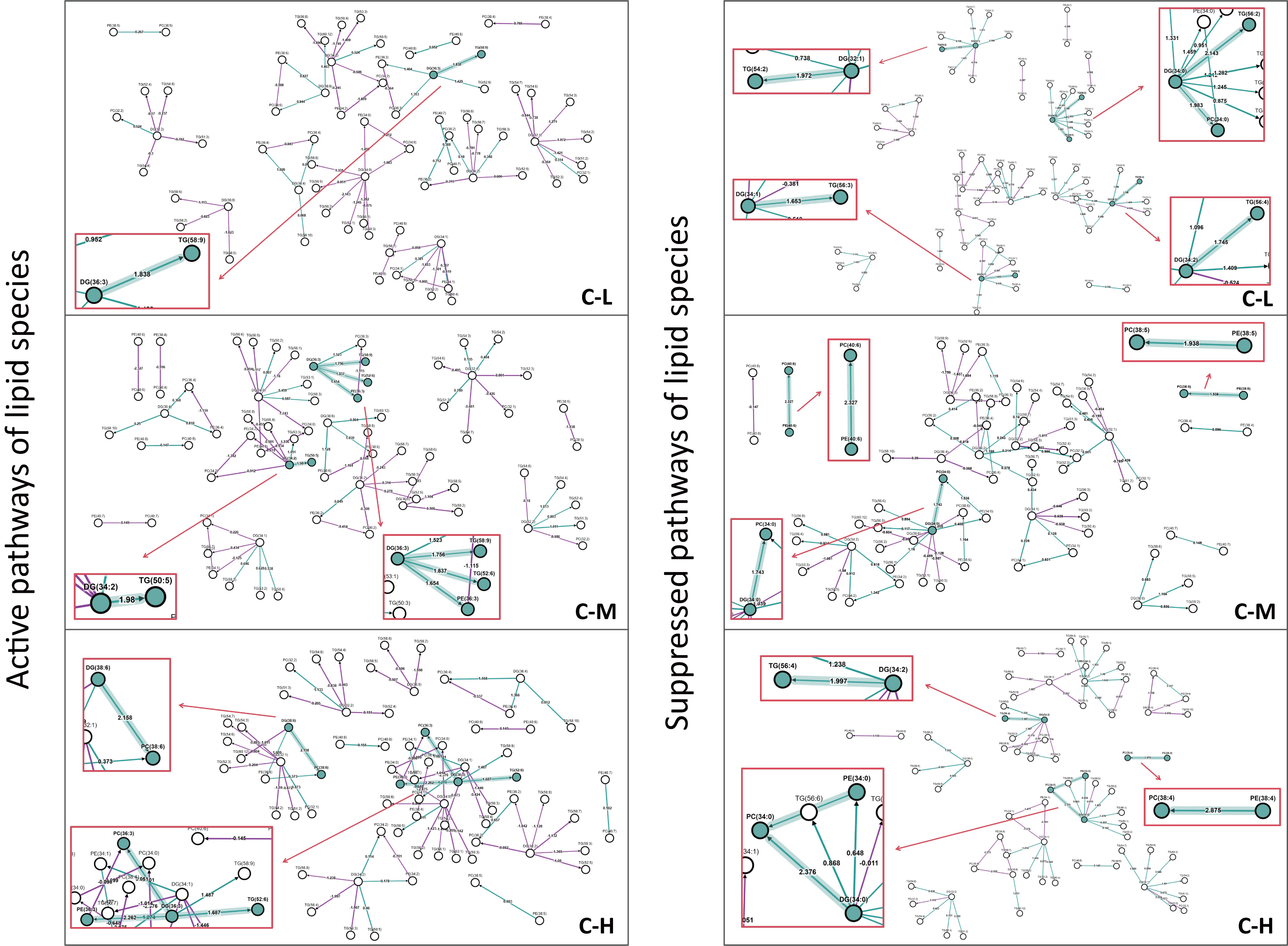

Supplement: Supplementary file 1 [file foods-14-02504-s001.zip › S1.png]

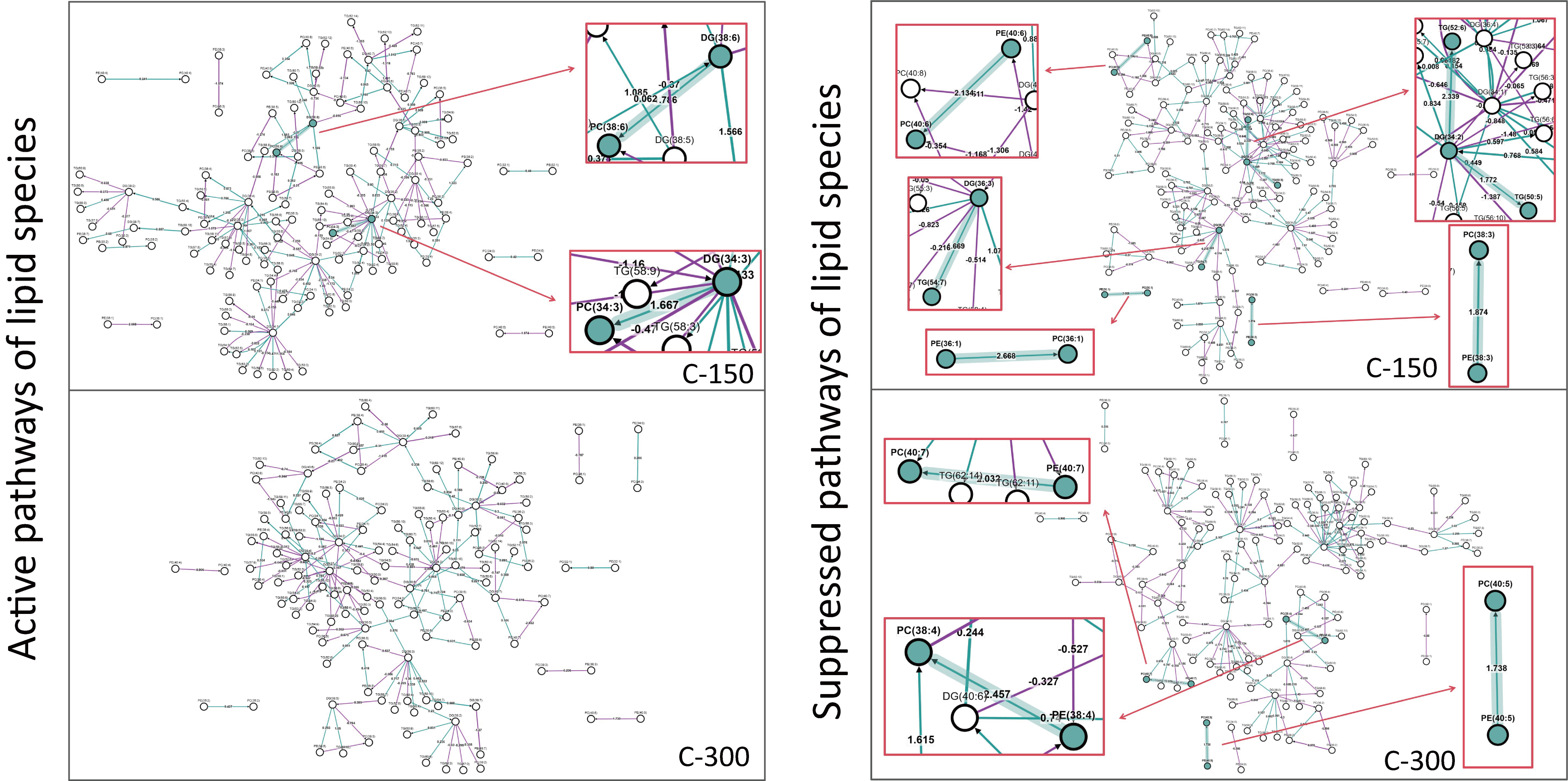

Supplement: Supplementary file 1 [file foods-14-02504-s001.zip › S2.png]
